# Insufficient expression of COL6A1 promotes the development of early-onset severe preeclampsia by inhibiting the APJ/AKT signaling pathway

**DOI:** 10.1038/s41420-025-02373-4

**Published:** 2025-03-01

**Authors:** Gonghua Qi, Yanmin Gong, Yi Li, Yanhui Jin, Shuqi Chi, Wenxia Zhang, Xia Luo

**Affiliations:** 1 Department of Obstetrics and Gynecology, Qilu Hospital, Shandong University, Jinan, China; 2 Gynecologic Oncology Key Laboratory of Shandong Province, Qilu Hospital, Shandong University, Jinan, China

**Keywords:** Cell invasion, Extracellular matrix

## Abstract

Early-onset severe preeclampsia (eosPE) is one of the most severe complications of pregnancy. To identify the genes related to the development of eosPE. We downloaded and integrated analyzed microarray data from GSE44711, GSE66273, and GSE74341, which contains the expression profile of placental tissues from patients with eosPE and healthy controls. Our analysis revealed that collagen type VI alpha 1 (COL6A1) was downregulated in the eosPE placenta compared to normal pregnancy. COL6A1 promoted the migration, invasion and tube formation ability of HTR8/SVneo cells, HUVECs and primary extravillous trophoblasts (EVTs). To explore the underlying mechanisms, we conducted transcriptome sequencing, which indicated that the Apelin/APJ signaling pathway was affected by COL6A1 knockdown. In addition, we found that APJ expression was lower in the placental tissue of patients with eosPE compared to healthy pregnancies. Inhibition of APJ suppressed the invasion, migration, and tube formation abilities of trophoblasts. We also observed that COL6A1 increased the levels of p-AKT and p-mTOR, while the APJ inhibitor ML221 impaired this effect. Furthermore, transwell and tube formation assays demonstrated that ML221 attenuated the capabilities enhanced by COL6A1, an effect that could be rescued by the AKT activator SC79. Overall, these findings indicate that insufficient expression of COL6A1 attenuates the migration, invasion, and endothelial-like tube formation of HTR8/SVneo cells and primary EVTs via the APJ/AKT/mTOR pathway, thereby promoting the development of eosPE.

## Introduction

Preeclampsia (PE) is one of the most severe complications during pregnancy and ranks as the second leading cause of maternal and perinatal mortality worldwide [1, 2]. Early-onset severe preeclampsia (eosPE) presents with more severe clinical manifestations, putting mothers at increased risk of multi-organ dysfunction and fetuses at risk for growth restriction, intrauterine distress, or even intrauterine fetal demise [3]. The earlier the onset, the poorer the prognosis.

Current research indicates that eoPE is primarily related to inadequate trophoblastic invasion, impaired remodeling of uterine spiral arteries, ischemia-reperfusion injury of the placenta, and oxidative stress in trophoblasts [4]. However, the pathogenesis of eosPE remains poorly understood. Investigating and elucidating its mechanisms is essential for reducing adverse outcomes for mothers and infants and improving overall prognosis.

To explore the potential pathogenesis mechanisms of eosPE, we downloaded and analyzed microarray data of patients with eosPE and healthy controls in GEO database (Table 1). Our analysis revealed significantly reduced transcriptional levels of human collagen type VI alpha 1 (COL6A1) in eosPE cases compared to normal controls. COL6A1 is a major extracellular matrix (ECM) protein that interacts with other ECM components to form a supportive network structure for cells [5]. Previous studies have indicated that COL6A1 promotes migration and invasion in various cancers, including lung cancer [6], renal cancer [7], prostate cancer [8], cervical cancer [9], and osteosarcoma [10]. However, the specific role and underlying mechanisms of COL6A1 in the development of eosPE remain unclear.

**Table 1:** Details of the microarrays used.

GEO series number	Healthy preterm control, *n*	Patients with EOPE, *n*	Tissues	Platform
GSE44711	8	8	Placenta	GPL10558
GSE66273	5	6	Placenta	GPL4133
GSE74341	5	7	Placenta	GPL16699

To clarify the specific molecular mechanism of COL6A1 in the development of eosPE, we conducted next-generation sequencing on HTR8/SVneo cells with COL6A1 knockdown. The sequencing results showed a significant reduction in the Apelin signaling pathway following COL6A1 knockdown. Apelin, also known as APLN, is the endogenous ligand of APJ (Angiotensin domain type 1 receptor-associated proteins), a member of the seven-transmembrane G protein-coupled receptors (GPCR) [11]. The Apelin/APJ system plays a crucial role in regulating angiogenesis and is closely associated with the pathogenesis of preeclampsia. This signaling pathway is involved in the regulation of placental functions, such as cell proliferation, apoptosis, endocrine activities, angiogenesis, and placental vascular tension [12]. Previous studies have demonstrated decreased expression of the apelin/APJ system in pre-eclamptic placentas [13–15]. Lingyu Ye et al. found that APJ expression was reduced in pre-eclamptic placentas and activated the PI3K/AKT pathway to protect trophoblast cells from oxidative stress damage [16]. However, the influence of COL6A1 on the PI3K/AKT pathway or its interaction with APJ has yet to be reported.

In this study, we investigated the expression of COL6A1 in eosPE placenta and explored its function in the migration, invasion and tube formation abilities of　HTR8/SVneo cells, HUVECs and primary EVTs. Furthermore, we clarified the involvement of the APJ/AKT/mTOR signaling pathway in the phenotypic changes induced by COL6A1.

## Results

### COL6A1 was downregulated in eosPE placentas and related to eosPE development

**Fig. 1:**
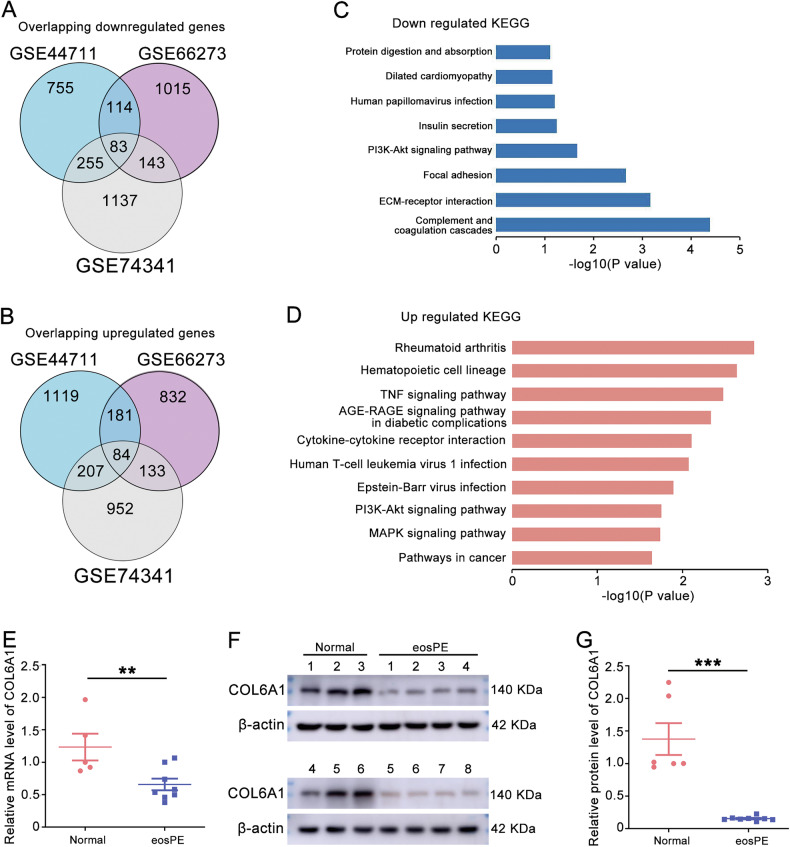
COL6A1 was downregulated in eosPE placentas. **A** The overlapping downregulated genes from datasets GSE44711, GSE66273, and GSE74341. **B** The overlapping upregulated genes from datasets GSE44711, GSE66273, and GSE74341. **C** KEGG pathway analysis of the overlapping downregulated genes in (**A**). **D** KEGG pathway analysis of overlapping upregulated genes in (**B**). **E** The mRNA level of COL6A1 in normal and eosPE placentas. **F**, **G** The protein levels of COL6A1 in normal and eosPE placentas (data are mean ± SD, **P < 0.01, ***P < 0.001, *n* = 3).

To identify genes related to the development of eosPE, we analyzed microarray data contain patients with eosPE and healthy controls from the GEO database (GSE44711, GSE66273 and GSE74341, Table 1). First, a total of 83 downregulated and 84 upregulated genes were identified (Fig. 1A, B). KEGG analysis showed the downregulated genes mainly participated in “Complement and coagulation cascades”, “ECM-receptor interaction”, “Focal adhesion” “PI3K-Akt signaling pathway” and “Insulin secretion” pathways (Fig. 1C, D). The PPI network of the overlapping DEGs suggested that SPP1, COL6A1, and COL6A2 were hub-downregulated genes, while KIT and LEP were hub-upregulated genes (Supplementary Figs. S1 and S2). Our patient samples showed that the mRNA level of COL6A1 was significantly downregulated in eosPE samples compared to healthy controls (Fig. 1E). In addition, COL6A1 protein level was also downregulated in eosPE placenta (Fig. 1F, G and original western blot Fig. 1A). These findings suggest that COL6A1 was downregulated in eosPE placenta and may play a pivotal role in the pathophysiology of eosPE.

### COL6A1 promoted the migration, invasion, and endothelial-like tube formation of HTR8/SVneo cells

**Fig. 2:**
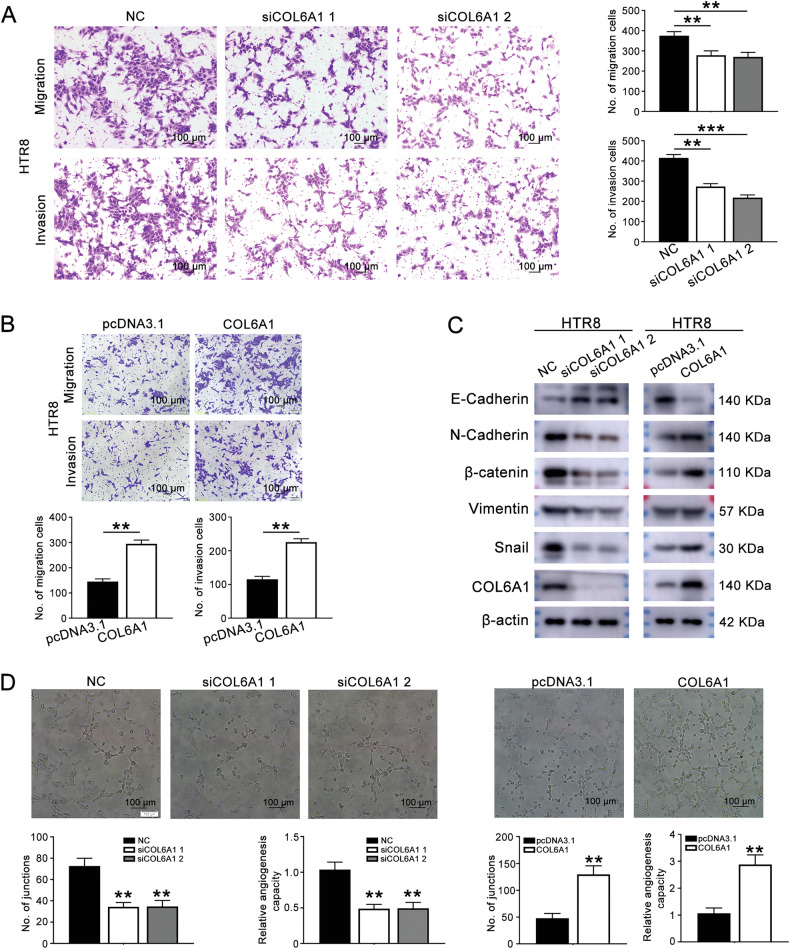
COL6A1 promoted the migration, invasion, and endothelial-like tube formation of HTR8/SVneo cells. **A** Transwell assay assessed the effect of COL6A1 knockdown on the migration and invasion of HTR8/SVneo cells (100×, scale bar: 100 μm). **B** The effect of COL6A1 overexpression on the migration and invasion of HTR8/SVneo cells was measured by transwell assay (100×, scale bar: 100 μm). **C** Western blot assay was performed to detect the EMT marker after COL6A1 knockdown or overexpression. **D** The effect of COL6A1 on endothelial-like tube formation of HTR8/SVneo cells (100×, scale bar: 100 μm) (data are mean ± SD, **P < 0.01, ***P < 0.001, *n* = 3).

To investigate the role of COL6A1 in the development of eosPE, COL6A1 knockdown and overexpression HTR8/SVneo cells were prepared (Supplementary Fig. S3A–D). MTT and colony formation assay showed that COL6A1 has no effect on the proliferation and colony formation abilities of HTR8/SVneo cells (Supplementary Fig. S3E–H). Transwell assays revealed that knockdown of COL6A1 inhibited the migration and invasion of the HTR8/SVneo cells (Fig. 2A). Conversely, overexpression of COL6A1 led to enhanced migration and invasion capabilities (Fig. 2B). Western blot analysis indicated that the expression of epithelial-to-mesenchymal transition (EMT) markers altered in response to changes in COL6A1 levels (Fig. 2C and original western blot Fig. 1B). In addition, endothelial-like tube formation assays showed that COL6A1 knockdown decreased the ability for endothelial-like tube formation, whereas overexpression of COL6A1 showed opposite effect (Fig. 2D). In summary, COL6A1 plays a crucial role in promoting the migration, invasion, and endothelial-like tube formation of HTR8/SVneo cells.

### Conditioned medium (CM) derived from COL6A1 knockdown/overexpressed HTR8/SVneo cells affected HUVECs functions

**Fig. 3:**
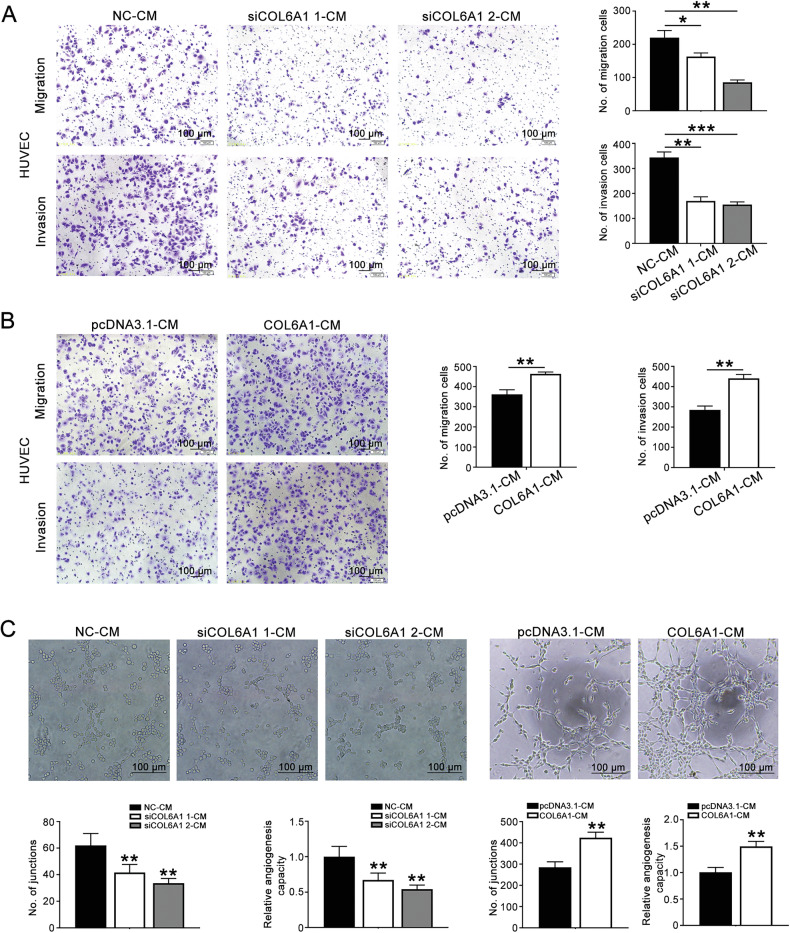
Conditioned medium (CM) derived from COL6A1 knockdown/overexpressed HTR8/SVneo cells affected HUVECs functions. CM derived from HTR8/SVneo cells transfected with an empty vector (pcDNA3.1) or COL6A1 overexpression plasmid referred to as pcDNA3.1-CM and COL6A1-CM. CM derived from HTR8/SVneo cells transfected with a negative control or COL6A1 siRNA was named NC-CM, siCOL6A1 1-CM, and siCOL6A1 2-CM. **A**, **B** HUVEC migration and invasion after treatment with CM was assessed by transwell assay (100×, scale bar: 100 μm). **C** Representative images and quantification of HUVEC tube formation after treatment with CM (100×, scale bar: 100 μm) (data are mean ± SD, *P < 0.05, **P < 0.01, ***P < 0.001, *n* = 3).

To investigate the influence of CM derived from COL6A1 knockdown or overexpressed HTR8/SVneo cells on HUVECs, we collected CM from these modified cells. Transwell assays revealed that treatment with siCOL6A1-CM significantly reduced the number of HUVECs migrating to the lower chamber compared to NC-CM (Fig. 3A). In contrast, HUVECs treated with COL6A1-CM exhibited increased migration and invasion (Fig. 3B). Moreover, tube formation assays indicated that siCOL6A1-CM treatment resulted in a decrease in the total junctions formed by HUVECs, whereas COL6A1-CM treatment enhanced the formation of junctions (Fig. 3C). These results collectively demonstrate that COL6A1-CM promotes the migration, invasion, and tube formation of HUVECs, highlighting the role of COL6A1 in modulating endothelial cell functions.

### Next-generation sequencing (NGS) revealed that Apelin signaling pathway was affected by COL6A1

**Fig. 4:**
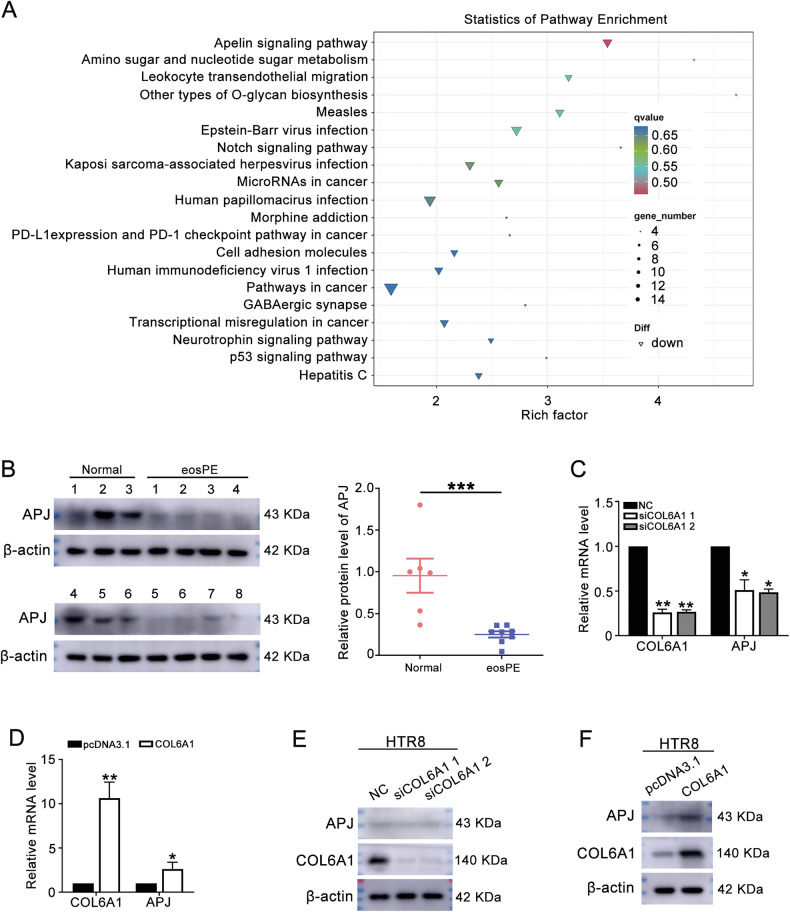
Next-generation sequencing (NGS) revealed that Apelin signaling pathway was affected by COL6A1. **A** NGS was performed on HTR8/SVneo cells transfected with siCOL6A1 1 or NC (*n* = 3) to analyze changes in the mRNA expression profile following COL6A1 knockdown. The KEGG pathway analysis of the downregulated DEGs was shown. **B** The protein level of APJ in normal and eosPE placenta. **C** The mRNA level of COL6A1 and APJ following COL6A1 knockdown. **D** HTR8/SVneo cells were transfected with COL6A1, and RT-qPCR was performed to detect the mRNA levels of COL6A1 and APJ. **E**, **F** The protein levels of COL6A1, APJ, and β-actin after COL6A1 knockdown or overexpression were measured by western blot (data are mean ± SD, *P < 0.05, **P < 0.01, ***P < 0.001, *n* = 3).

To elucidate the potential mechanisms underlying the phenotypic changes induced by COL6A1, NGS was performed on COL6A1-knockdown (siCOL6A1) and negative control (NC) HTR8/SVneo cells. This analysis identified a total of 501 genes, including 217 upregulated and 284 downregulated genes (Supplementary Fig. S4). KEGG pathway analysis indicated a significant downregulation of the Apelin signaling pathway (Fig. 4A). In addition, the protein level of APJ was lower in eosPE placenta compared to healthy control (Fig. 4B and original western blot Fig. 1C). Furthermore, both mRNA and protein levels of APJ decreased following COL6A1 knockdown and increased upon COL6A1 overexpression (Fig. 4C–F, Supplementary Fig. S5A, B, and original western blot Figs. 1D and 2A). From all above, knockdown COL6A1 inhibited, and COL6A1 overexpression activated the Apelin signaling pathway.

### APJ promoted the migration, invasion, and endothelial-like tube formation of HTR8/SVneo cells

**Fig. 5:**
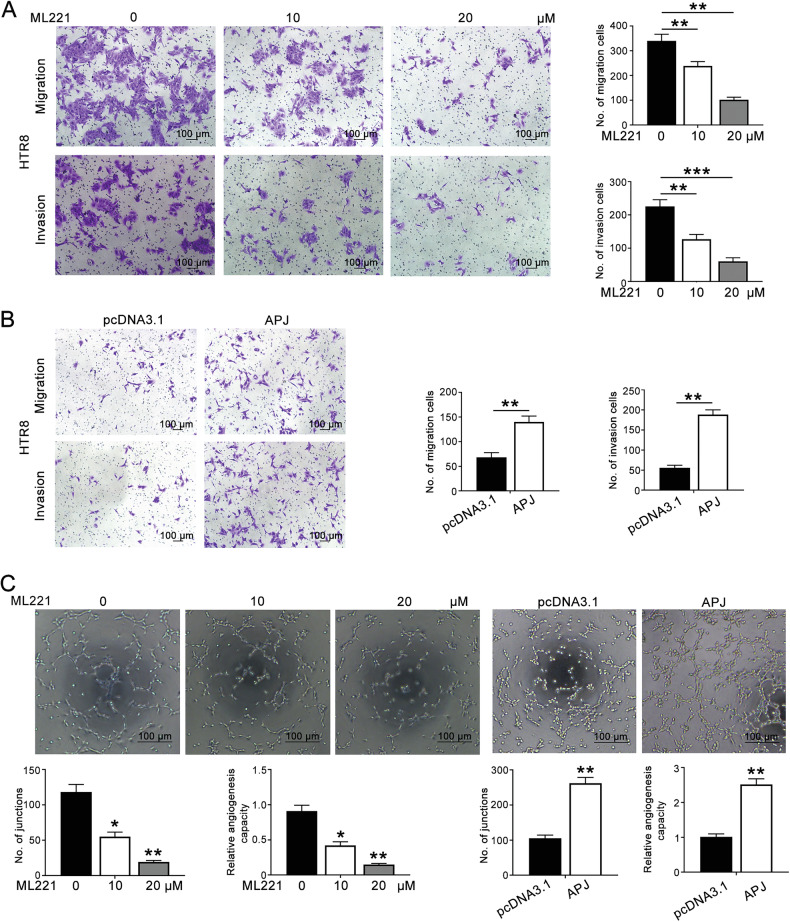
APJ promoted the migration, invasion, and endothelial-like tube formation of HTR8/SVneo cells. **A** Transwell assay was conducted to detect the migration and invasion of ML221-treated HTR8/SVneo cells (100×, scale bar: 100 μm). **B** The effects of APJ overexpression on the migration and invasion of HTR8/SVneo cells were measured (100×, scale bar: 100 μm). **C** The influence of APJ on endothelial-like tube formation in HTR8/SVneo cells was examined (100×, scale bar: 100 μm) (data are mean ± SD, *P < 0.05, **P < 0.01, ***P < 0.001, *n* = 3).

Next, we investigated the effects of APJ on the function of HTR8/SVneo cells. Treatment with ML221, an APJ inhibitor, significantly reduced both migration and invasion of HTR8/SVneo cells (Fig. 5A). Conversely, APJ overexpression led to an increase in the number of migrating and invading cells (Fig. 5B). Moreover, tube formation assays revealed that ML221 decreased while APJ overexpression enhanced the junctions formed by HTR8/SVneo cells (Fig. 5C). These results suggested that APJ plays a crucial role in promoting the migration, invasion, and tube formation of HTR8/SVneo cells.

### COL6A1 promoted the migration, invasion, and endothelial-like tube formation of HTR8/SVneo cells through APJ/AKT/mTOR pathway

**Fig. 6:**
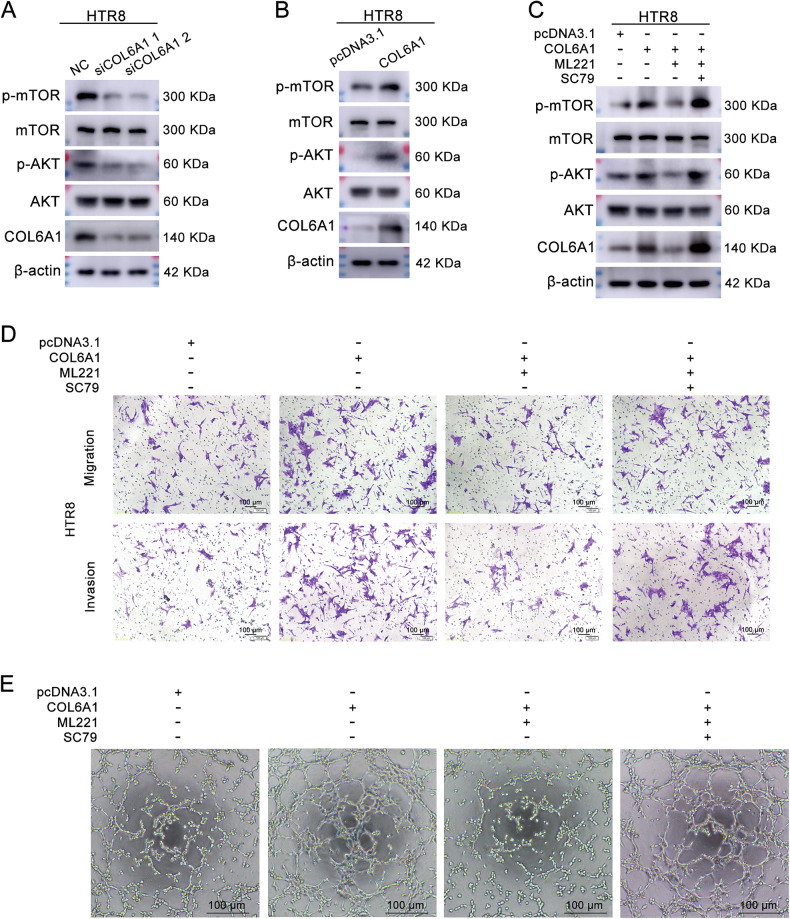
COL6A1 promoted the migration, invasion, and endothelial-like tube formation of HTR8/SVneo cells through APJ/AKT/mTOR pathway. **A**, **B** The protein levels of COL6A1, p-AKT, AKT, p-mTOR, mTOR, and β-actin following COL6A1 knockdown or overexpression were measured by western blot. **C**–**E** COL6A1-overexpressed HTR8/SVneo cells and their corresponding control cells were treated with ML221 alone or combined with SC79. The protein levels of COL6A1, p-AKT, AKT, p-mTOR, mTOR, and β-actin were detected by western blot (**C**). Transwell assay (**D**) and endothelial-like tube formation assay (**E**) were performed (100×, scale bar: 100 μm).

Previous research has indicated that APJ can activate the PI3K/AKT pathway, and we have demonstrated that COL6A1 activated APJ. However, it remains unclear whether COL6A1 influences the PI3K/AKT pathway and whether this effect occurs through APJ. Firstly, western blot assay showed that COL6A1 knockdown decreased the level of p-AKT and p-mTOR (Fig. 6A, Supplementary Fig. S6A, and original western blot Fig. 2B), whereas COL6A1 overexpression increased these levels (Fig. 6B, Supplementary Fig. S6B, and original western blot Fig. 2C). Subsequently, HTR8/SVneo cells overexpressing COL6A1 and their corresponding controls were treated with or without ML221. Western blot analysis indicated that ML221 treatment diminished the p-AKT and p-mTOR levels induced by COL6A1 (Fig. 6C, Supplementary Fig. S6C, and original western blot Fig. 2D). Furthermore, transwell and tube formation assays revealed that ML221 attenuated the migration and tube formation capabilities enhanced by COL6A1 overexpression. Notably, this inhibitory effect could be rescued by the AKT activator SC79 (Fig. 6D, E and Supplementary Fig. S6D, E). These findings revealed that COL6A1 promoted the migration, invasion, and endothelial-like tube formation of HTR8/SVneo cells through the APJ/AKT/mTOR pathway.

### COL6A1 promoted the migration and invasion of primary EVTs through the APJ/AKT/mTOR pathway

**Fig. 7:**
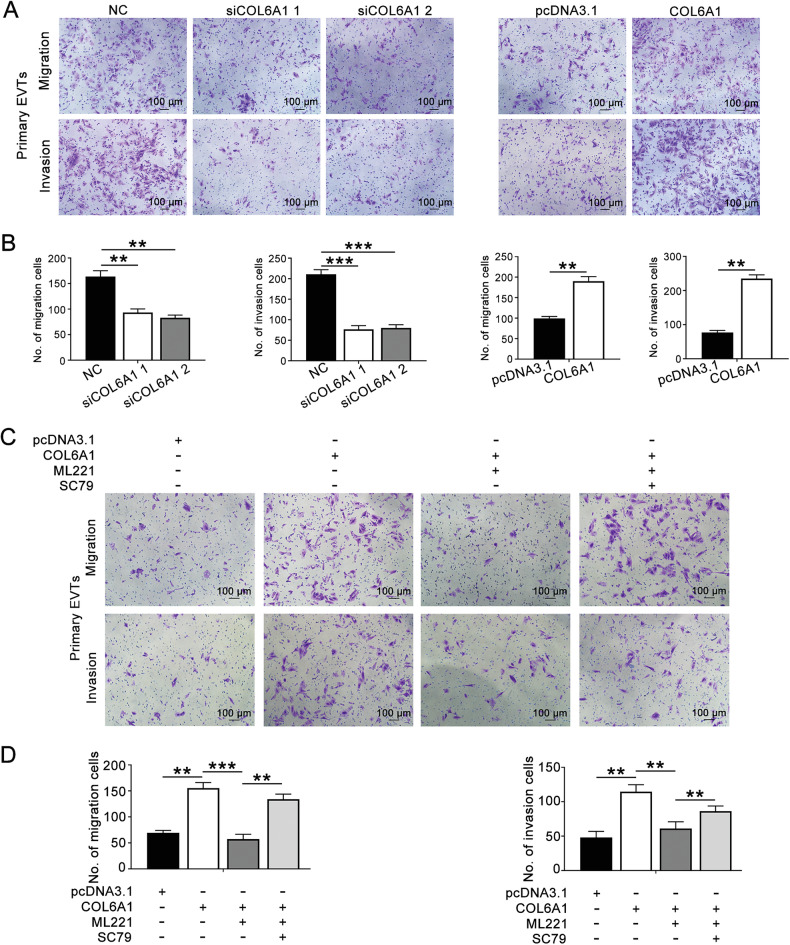
COL6A1 promoted the migration and invasion of primary EVTs through the APJ/AKT/mTOR pathway. **A,** **B** Transwell assays were conducted to detect the effect of COL6A1 on the migration and invasion abilities of primary EVTs (100×, scale bar: 100 μm). **C**, **D** Primary EVTs were firstly transfected with pcDNA3.1 or COL6A1, and ML221 was added alone or in combination with SC79 for 24 h. Transwell assays were performed to measure changes in migration and invasion (100×, scale bar: 100 μm) (data are mean ± SD, **P < 0.01, ***P < 0.001, *n* = 3).

To further validate our findings, primary human EVTs were isolated and cultured. Transwell assays demonstrated that knockdown of COL6A1 significantly inhibited the migration and invasion of primary EVTs, while overexpression of COL6A1 enhanced these abilities (Fig. 7A, B). In addition, ML221 attenuated the increased migration and invasion abilities induced by COL6A1 overexpression. This effect was reversible by the AKT activator SC79 (Fig. 7C, D). These results provide strong evidence that COL6A1 promotes the migration and invasion of primary EVT cells through the APJ/AKT/mTOR pathway.

## Discussion

In this study, we employed an integrated method to investigate potential genes related to the development of eosPE. We first analyzed and downloaded microarray data from GSE44711, GSE66273, and GSE74341, which contain the expression profile of placental tissues from patients with eosPE and healthy controls. He et al. [17], Ma et al. [18], and Song et al. [19] reanalyzed the microarray data from GSE44711, while Zhang et al. [20] performed an integrated analysis of GSE44711 and GSE74341 to identify DEGs and partially validated by RT-qPCR. In this study, we conducted a comprehensive analysis of three microarrays, enhancing the strength and validity of our findings.

In the current research, a total of 83 overlapping downregulated and 84 overlapping upregulated genes were identified. KEGG analysis of the downregulated DEGs aligned with those of Zhang et al. [20]. We also constructed a PPI network and analyzed hub genes. The top three downregulated hub genes (SPP1, COL6A1, and COL6A2) and the top two upregulated hub genes (KIT and LEP) were further analyzed and validated. SPP1 has been identified as a key gene related to the eoPE [21–23]. In addition, Serum LEP levels have been found to be higher in patients with PE [24] and elevated in patients with eoPE compared to those with loPE [25]. KIT [22] and COL6A1 [26] have been reported to be associated with PE through bioinformatics methods, however, they have not yet been verified using clinical samples. We used patients’ samples and found that COL6A1 was significantly lower in eosPE compared to normal pregnancies.

COL6A1, a major ECM protein, has been implicated in promoting migration and invasion in various cancers [6–10]. However, its specific role and underlying mechanisms in the development of eosPE have not been fully elucidated. In the present study, we demonstrated that knocking down COL6A1 significantly impaired the migration, invasion, and tube formation abilities of HTR8/SVneo cells, HUVECs and primary EVTs. These findings suggest that COL6A1 plays a crucial role in trophoblast function and may be an important gene associated with the etiology of PE.

To elucidate the mechanisms underlying the effects of COL6A1, we conducted transcriptome sequencing on HTR8/SVneo cells with COL6A1 knockdown. Apelin signaling pathway was affected by COL6A1 knockdown. The Apelin/APJ signaling pathway is involved in the regulation of placental functions, including cell proliferation, apoptosis, endocrine activities, angiogenesis, and placental vascular tension [12]. We observed that APJ expression was significantly lower in the placental tissue of patients with eosPE compared to healthy pregnancies, which corroborate previous studies [13–15]. Furthermore, our experiments demonstrated that the knockdown of APJ led to inhibited invasion, migration, and tube formation abilities of trophoblasts.

Lingyu Ye et al. reported that APJ expression was reduced in pre-eclamptic placentas and activated the PI3K/AKT pathway to protect trophoblast cells from oxidative stress damage [16]. However, the relationship between COL6A1 and the PI3K/AKT pathway, particularly whether COL6A1 influences this pathway via APJ, has not been previously explored. In the present study, we found that COL6A1 increased the levels of p-AKT and p-mTOR. Notably, the administration of the APJ inhibitor ML221 impaired this effect, indicating a crucial role for the APJ signaling pathway in mediating COL6A1’s actions. Furthermore, transwell and tube formation assays demonstrated that ML221 attenuated the increased migratory and invasive abilities induced by COL6A1 overexpression. Importantly, this impairment could be rescued by the AKT activator SC79, reinforcing the notion that COL6A1 promotes these cellular processes through the APJ/AKT/mTOR signaling pathway.

These findings suggest that COL6A1 enhances the migration, invasion, and endothelial-like tube formation of HTR8/SVneo cells by activating the APJ/AKT/mTOR pathway, shedding light on potential therapeutic targets for managing eosPE. Further investigations are warranted to explore the broader implications of this signaling axis in the context of patients with eosPE and healthy controls.

## Materials and methods

### Bioinformatics analysis

GSE44711, GSE66273, and GSE74341, which contain microarray data from patients with eosPE and healthy controls are included (Table 1).

The differentially expressed genes (DEGs) were analyzed by GEO2R with an adjusted *P* value < 0.05 and fold change >1.5. The overlapping DEGs were identified using Venny 2.1 (https://bioinfogp.cnb.csic.es/tools/venny/). Kyoto Encyclopedia of Genes and Genomes (KEGG) pathway analysis of the DEGs was performed using DAVID (https://david.ncifcrf.gov/tools.jsp). Additionally, the Protein-Protein Interaction (PPI) network for DEGs was analyzed using STRING (http://string-db.org).

### Patients and tissue samples

This research was approved by the Ethics Committee at Qilu Hospital of Shandong University. Placental samples were collected from pregnant women who gave birth at Qilu Hospital between January 2023 and August 2024. The diagnosis of eosPE was based on criteria set forth by the American College of Obstetricians and Gynecologists criteria [27]. Healthy pregnant women without complications served as controls. Following delivery, placental tissues were immediately collected, rinsed, and stored at −80 °C. All participants provided informed consent.

### Cell culture

The HTR8/SVneo cell line was obtained from Shanghai Enzyme Research Biotechnology Co. and cultured in RPMI-1640 with 10% fetal bovine serum (FBS). The human umbilical vein endothelial cells (HUVECs) were generously provided by JunYing Miao’s laboratory and maintained in DMEM with 10% FBS. Human primary EVTs were isolated from chorionic villous tissues and cultured in DMEM/F12 containing 10% FBS (The detailed steps are provided in the supplementary materials, all materials from Gibco, Grand Island, NY, USA). All cells were incubated at 37 °C in a 5% CO2 atmosphere.

### RNA interference and overexpression

The knockdown and overexpression of COL6A1 was performed using Lipofectamine 2000 (11668-019, Invitrogen) according to the manufacturer’s instructions. Details are shown in the supplementary materials.

### RNA isolation and RT-qPCR

RNA isolation and RT-qPCR were performed, as previously described [28]. The primer sequences are provided in the supplementary materials.

### Protein extraction and western blotting

Protein extraction and western blotting were performed as previously described [28]. The primary antibodies are shown below: COL6A1 (1:1000, Proteintech, 17023-1-AP), APJ (1:1000, Sangon Biotech, D120227), p-mTOR (1:1000, CST, 5536T), mTOR (1:1000, Servicebio, GB11405), p-Akt (1:1000, Abcam, ab81283), Akt (1:1000, Proteintech, 60203-2-Ig), β-actin (1:5000, Sigma-Aldrich, A5441).

### Preparation of conditioned medium (CM)

The medium of COL6A1 knockdown and overexpression HTR8/SVneo cells was replaced with serum-free DMEM. Forty-eight hours later, the conditioned medium (CM) was collected, centrifuged at 2000 rpm for 10 minutes, filtered through a 0.22-μm filter, and then frozen at −80 °C.

### Tube formation assay

Matrigel (Corning, 356231) was added to each well of 96-well plates and incubated at 37 °C for 1 h. Then, 5 × 10^4^ transfected HTR8/SVneo cells or CM-treated HUVECs (24 h) were suspended in 100 μL of the corresponding medium and seeded into each well. After incubating for another 6 h, images of each well were captured. Finally, the angiogenesis analysis tool in ImageJ was used to measure the junctions of the tubes.

### Statistical analysis

The data were presented as the means ± SDs. Student’s *t* test and one-way ANOVA in SPSS v22.0 (SPSS, Inc., Chicago, IL, USA) were used to analyze the significance between two or more groups, respectively. A *P* value of <0.05 was considered statistically significant (^#^*P* > 0.05, *P < 0.05, **P < 0.01, ***P < 0.001). GraphPad Prism 8.00 (GraphPad Software, La Jolla, CA, USA) and Adobe Photoshop CC 2019 (Adobe, San Jose, CA, USA) were utilized for data and graphic processing. Every experiment was repeated independently at least three times.

## Supplementary information

Original figures of western blo

Supplementary materials

## Data Availability

All data generated or analyzed during this study are included in this published article.

## References

[1] Espinoza J, Vidaeff A, Pettker CM, Simhan H. Gestational hypertension and preeclampsia. Obstet Gynecol. 2020;135:E237–60. 10.1097/AOG.0000000000003891 32443079

[2] Brown MA, Magee LA, Kenny LC, Karumanchi SA, McCarthy FP, Saito S, et al. The hypertensive disorders of pregnancy: ISSHP classification, diagnosis & management recommendations for international practice. Pregnancy Hypertens. 2018;13:291–310. 29803330 10.1016/j.preghy.2018.05.004

[3] Reinaldo M, Delia IC, Cilia A, Deliana R, Fernando T, Luis S. Oxidative stress and mitochondrial dysfunction in early-onset and late-onset preeclampsia. Biochim Biophys Acta Mol Basis Dis. 2020;1866:165961. 32916282 10.1016/j.bbadis.2020.165961

[4] Dahlia R, Erika P. A critical review of early-onset and late-onset preeclampsia. Obstet Gynecol Surv. 2011;66:497–506. 22018452 10.1097/OGX.0b013e3182331028

[5] Keene DR, Engvall E, Glanville RW. Ultrastructure of type VI collagen in human skin and cartilage suggests an anchoring function for this filamentous network. J Cell Biol. 1988;107:1995–2006. 3182942 10.1083/jcb.107.5.1995 PMC2115316

[6] Kuo-Hsun C, Ying-Hwa C, Yu-Shun W, Shu-Hui L, Pao-Chi L. Quantitative secretome analysis reveals that COL6A1 is a metastasis-associated protein using stacking gel-aided purification combined with iTRAQ labeling. J Proteome Res. 2010;10:1110–25. 10.1021/pr1008724 21186846

[7] Fangning W, Hongkai W, Yijun S, Hailiang Z, Guohai S, Yao Z, et al. Upregulation of COL6A1 is predictive of poor prognosis in clear cell renal cell carcinoma patients. Oncotarget. 2015;6:27378–87. 26317545 10.18632/oncotarget.4860 PMC4694996

[8] Yi-Ping Z, Fang-Ning W, Yi-Jun S, Hong-Kai W, Gui-Ming Z, Ding-Wei Y. Reactive stroma component COL6A1 is upregulated in castration-resistant prostate cancer and promotes tumor growth. Oncotarget. 2015;6:14488–96. 25895032 10.18632/oncotarget.3697 PMC4546481

[9] Teng H, Chongjie T, Gallina K, Weijing Z, Xin H, Yongwen H, et al. Expression of COL6A1 predicts prognosis in cervical cancer patients. Am J Transl Res. 2016;8:2838–44. 27398167 PMC4931178

[10] Ying Z, Zhaoyong L, Xia Y, Weiqing L, Yelong C, Youbin L, et al. H3K27 acetylation activated-COL6A1 promotes osteosarcoma lung metastasis by repressing STAT1 and activating pulmonary cancer-associated fibroblasts. Theranostics. 2021;11:1473–92. 33391546 10.7150/thno.51245 PMC7738898

[11] Feng X, Deguan L, Linxi C. ELABELA: a novel hormone in cardiac development acting as a new endogenous ligand for the APJ receptor. Acta Biochim Biophys Sin. 2014;46:620–2. 24829400 10.1093/abbs/gmu032

[12] Dawid M, Mlyczyńska E, Jurek M, Respekta N, Pich K, Kurowska P, et al. Apelin, APJ, and ELABELA: role in placental function, pregnancy, and foetal development—an overview. Cells. 2021;11:99. 35011661 10.3390/cells11010099 PMC8750556

[13] Furuya M, Okuda M, Usui H, Takenouchi T, Kami D, Nozawa A, et al. Expression of angiotensin II receptor-like 1 in the placentas of pregnancy-induced hypertension. Int J Gynecol Pathol. 2012;31:227–35. 22498939 10.1097/PGP.0b013e31823b6e71

[14] Inuzuka H, Nishizawa H, Inagaki A, Suzuki M, Ota S, Miyamura H, et al. Decreased expression of apelin in placentas from severe pre-eclampsia patients. Hypertension Pregnancy. 2013;32:410–21. 10.3109/10641955.2013.813535 23844873

[15] Simsek Y, Celik O, Yilmaz E, Karaer A, Dogan C, Aydin S, et al. Serum levels of apelin, salusin-alpha and salusin-beta in normal pregnancy and preeclampsia. J Matern-fetal neonatal Med. 2012;25:1705–8. 22533552 10.3109/14767058.2011.660221

[16] Ye L, Huang Y, Liu X, Zhang X, Cao Y, Kong X, et al. Apelin/APJ system protects placental trophoblasts from hypoxia-induced oxidative stress through activating PI3K/Akt signaling pathway in preeclampsia. Free Radic Biol Med. 2023;208:759–70. 37774802 10.1016/j.freeradbiomed.2023.09.030

[17] He P, Shao D, Ye M, Zhang G. Analysis of gene expression identifies candidate markers and pathways in pre-eclampsia. J Obstet Gynaecol. 2014;35:578–84. 25528892 10.3109/01443615.2014.990430

[18] Yifei M, Hongmei L, Hong Z, Xiu’e S, Huili YJJOGR. Identification of potential crucial genes associated with early-onset pre-eclampsia via a microarray analysis. J Obstet Gynaecol Res. 2017;43:812–9. 28759171 10.1111/jog.13275

[19] Song J, Li Y, An RF. Identification of early-onset preeclampsia-related genes and microRNAs by bioinformatics approaches. Reprod Sci. 2015;22:954–63. 25717061 10.1177/1933719115570898

[20] Hao Z, Lu X, Yan L, Xiang Y, Yiwei Z, Zhijing M, et al. Integrated microarray analysis of key genes and a miRNA‑mRNA regulatory network of early‑onset preeclampsia. Mol Med Rep. 2020;22:4772–82. 10.3892/mmr.2020.11551 PMC7646902 33173953

[21] Kang Q, Li W, Xiao J, Yu N, Fan L, Sha M, et al. Identification of potential crucial genes associated with early-onset preeclampsia via bioinformatic analysis. Pregnancy Hypertens. 2021;24:27–36. 33640831 10.1016/j.preghy.2021.02.007

[22] Zhang Z, Wang P, Zhang L, Huang C, Gao J, Li Y, et al. Identification of key genes and long noncoding RNA-associated competing endogenous RNA (ceRNA) networks in early-onset preeclampsia. Biomed Res Int. 2020;2020:1673486. 32566660 10.1155/2020/1673486 PMC7293732

[23] Zhan F, He L, Wu J, Wu XJBG. Bioinformatic analysis identifies potential extracellular matrix related genes in the pathogenesis of early onset preeclampsia. Biochem Genet. 2024;62:646–65. 37498421 10.1007/s10528-023-10461-2

[24] Taylor B, Ness R, Olsen J, Hougaard D, Skogstrand K, Roberts J, et al. Serum leptin measured in early pregnancy is higher in women with preeclampsia compared with normotensive pregnant women. Hypertension. 2015;65:594–9. 25510827 10.1161/HYPERTENSIONAHA.114.03979 PMC4326535

[25] Salimi S, Farajian-Mashhadi F, Naghavi A, Mokhtari M, Shahrakipour M, Saravani M, et al. Different profile of serum leptin between early onset and late onset preeclampsia. Dis Markers. 2014;2014:628476. 24591763 10.1155/2014/628476 PMC3925616

[26] He P, Shao D, Ye M, Zhang G. Analysis of gene expression identifies candidate markers and pathways in pre-eclampsia. J Obstetr Gynaecol. 2015;35:578–84. 10.3109/01443615.2014.990430 25528892

[27] Obstetricians ACo G. Hypertension in pregnancy. Report of the American College of Obstetricians and Gynecologists’ Task Force on Hypertension in Pregnancy. Obstet Gynecol. 2013;122:122. 10.1097/01.AOG.0000437382.03963.88 24150027

[28] Qi G, Ma H, Li Y, Peng J, Chen J, Kong BJCd, et al. TTK inhibition increases cisplatin sensitivity in high-grade serous ovarian carcinoma through the mTOR/autophagy pathway. Cell Death Dis. 2021;12:1135. 34876569 10.1038/s41419-021-04429-6 PMC8651821

