# Supplementary material for: Insufficient expression of COL6A1 promotes the development of early-onset severe preeclampsia by inhibiting the APJ/AKT signaling pathway
Source: Cell Death Discov. 2025 Mar 1;11:81. doi: 10.1038/s41420-025-02373-4 (PMC11873267; doi:10.1038/s41420-025-02373-4)
Supplement: Supplementary file 1 — Original figures of western blo [file 41420_2025_2373_MOESM1_ESM.docx]

**Original western blot figures and figure legends**:

**Original western blot figure legends**:

Original western blot Figure 1. Original, full-length blot images of Figure 1 to Figure 4E.

A. Original blot images of Figure 1F.

B. Original blot images of Figure 2C.

C. Original blot images of Figure 4B.

D. Original blot images of Figure 4E.

Original western blot Figure 2. Original, full-length blot images of Figure 4F-6B.

A. Original, full-length blot images of Figure 4F.

B. Original, full-length blot images of Figure 6A.

C. Original, full-length blot images of Figure 6B.

D. Original, full-length blot images of Figure 6C.

**Original western blot Figures：**

**Original western blot Figure 1**

**
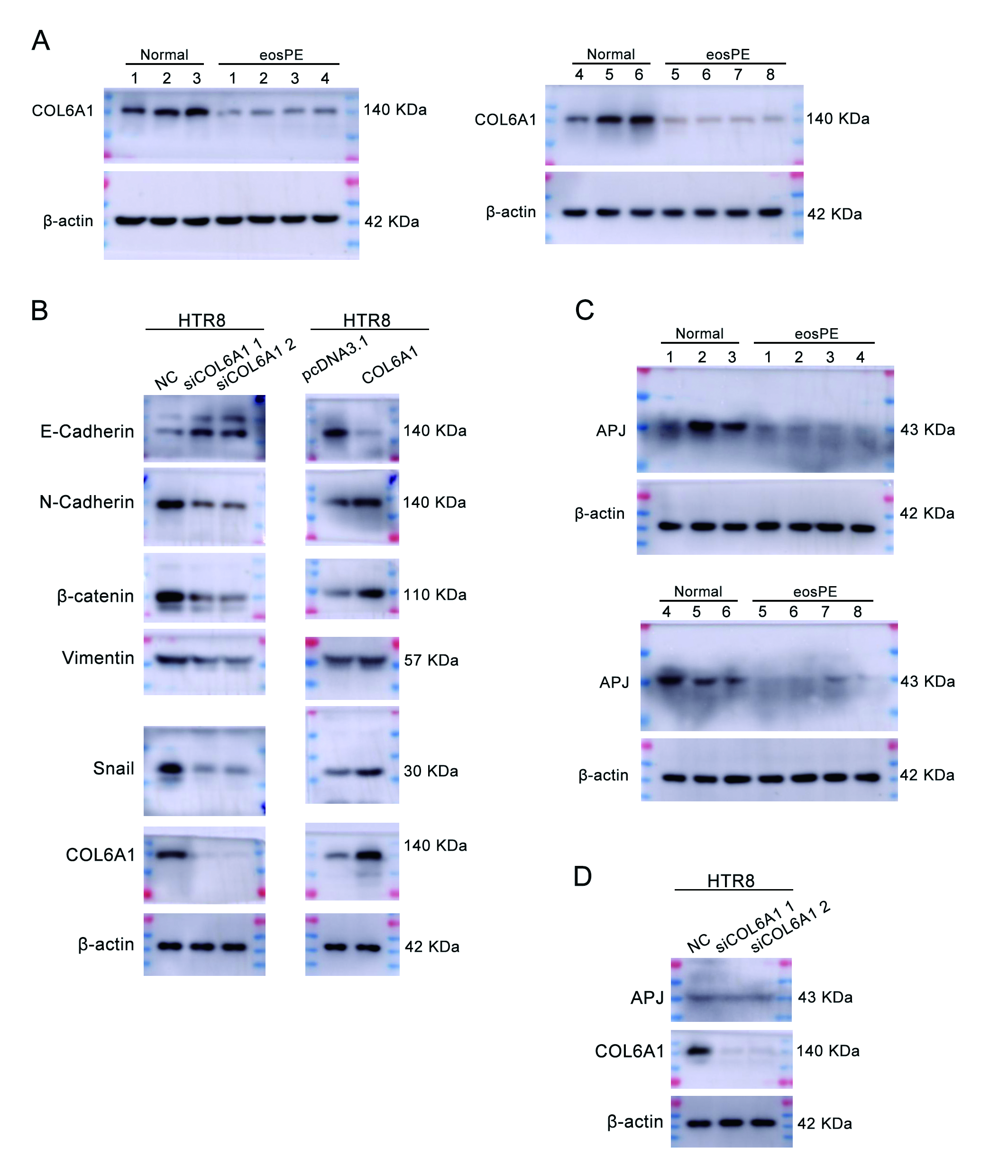
**

**Original western blot Figure 2**

**
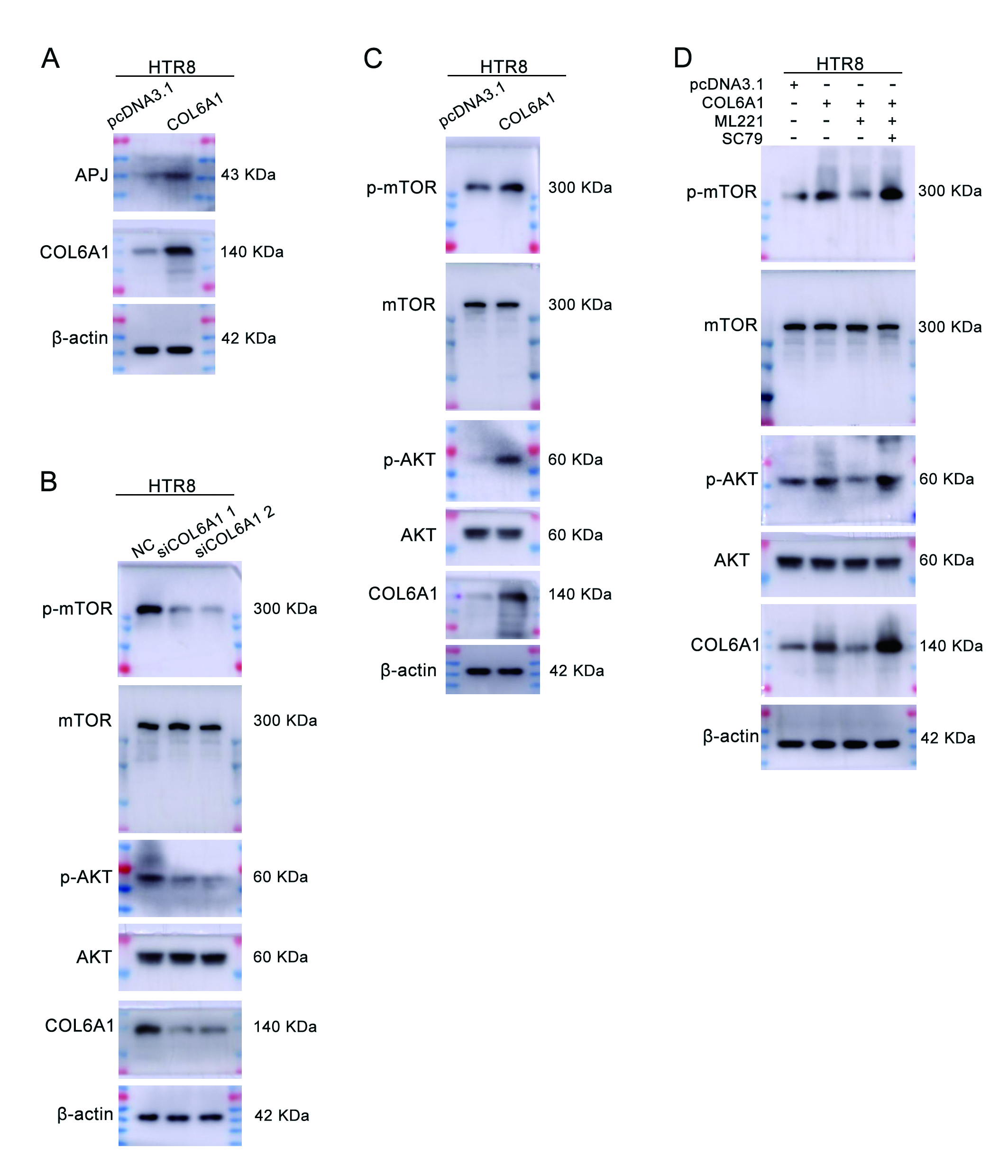
**
