# Supplementary material for: Insufficient expression of COL6A1 promotes the development of early-onset severe preeclampsia by inhibiting the APJ/AKT signaling pathway
Source: Cell Death Discov. 2025 Mar 1;11:81. doi: 10.1038/s41420-025-02373-4 (PMC11873267; doi:10.1038/s41420-025-02373-4)
Supplement: Supplementary file 2 — Supplementary materials [file 41420_2025_2373_MOESM2_ESM.docx]

**Supplementary Materials and Methods**

**Human primary EVTs isolation and culture**

Human primary EVTs were isolated from chorionic villous tissues collected during artificial abortion via vacuum aspiration (6-8 weeks gestation). First, the chorionic villous tissues were cleaned and dissected into 1-2 mm fragments. The fragments were then digested with 0.125% trypsin (including 0.02% EDTA) and type I collagenase (1 mg/mL) at 37 ℃ for 30 min, stopped with DMEM medium with 10% FBS. Next, the supernatant was centrifuged at 1000 rpm for 10 minutes, and the pellet was resuspended in serum-free DMEM. The cells were purified using a Percoll gradient, collecting the fraction between 60% and 35% Percoll. These cells were washed twice and resuspended in DMEM/F12 containing 10% FBS at a concentration of 1×10^5^ cells/mL. Finally, the cells were cultured in 6-well plate and passaged every 3 days.

**RNA interference and overexpression**

To knockdown the expression of COL6A1, specific siRNA targeted COL6A1 was synthesized by GenePharma (Shanghai, China). The COL6A1 siRNA and the negative control (NC) were transfected into HTR-8/SVneo cells at 50% confluence using Lipofectamine 2000 (11668-019, Invitrogen) according to the manufacturer’s instructions. For overexpression studies, the COL6A1 plasmid and the corresponding control vector (pcDNA3.1) were purchased from YouBio (Hunan, China). Cells at 80% confluence were transfected with the COL6A1 or pcDNA3.1 plasmid using Lipofectamine 2000.

**Primer sequences for RT–qPCR**

COL6A1 F: GCCTTCCTGAAGAATGTCACCG, R: TCCAGCAGGATGGTGATGTCAG. APJ F: ACTTCCGCAAGGAACGCATCGA, R: ACAGCGTCTTCACCAGGTGGTA. β-actin F: CACCATTGGCAATGAGCGGTTC, R: AGGTCTTTGCGGATGTCCACGT.

**Transwell assays**

The transwell assays were performed as previously described(1).

**Cell proliferation assay**

One thousand HTR8/SVneo cells were seeded into each well of 96-well plates and cultured for 5 days. Each day, 20 μL of MTT (Sigma-Aldrich, USA) was added to each well and incubated for an additional 4 hours. After incubation, the supernatant was discarded and 100 μL of DMSO (Sigma-Aldrich, USA) was added. Finally, the absorbance value at 490 nm was measured using a Varioskan Flash microplate reader (Thermo Scientific).

**Colony formation assay**

A total of 1000 transfected HTR8/SVneo cells were seeded into each well of 6-well plates. After incubating for 10-14 days, the cells were fixed with methanol and stained with crystal violet. Images were then captured, and the number of colonies was counted.

**Supplementary figures and figure legends:**

**Supplementary figure legends：**

**Supplementary Figure S1. Protein-Protein Interaction (PPI) network of the down-regulated DEGs**

**Supplementary Figure S2. Protein-Protein Interaction (****PPI) network of the up-regulated DEGs**

**Supplementary Figure S3. The effect of COL6A1 on the proliferation of HTR8/SVneo cells**

A and B. HTR8/SVneo cells were transfected with either siCOL6A1 1 or a negative control (NC). (A) The mRNA levels of COL6A1 were assesed by RT–qPCR. (B) Western blot analysis was conducted to evaluate the protein levels of COL6A1 and β-actin following COL6A1 knockdown.

C and D. HTR8/SVneo cells were transfected with either pcDNA3.1 or COL6A1. (C) RT–qPCR was employed to measure the mRNA levels of COL6A1. (D) The protein levels of COL6A1 and β-actin were determined through Western blot analysis.

E and F. MTT assays were performed to assess the impact of COL6A1 knockdown or overexpression on the proliferation of HTR8/SVneo cells.

G and H. The colony formation ability of HTR8/SVneo cells with COL6A1 knockdown or overexpression was evaluated using a colony formation assay.

(Data are mean ± SD, ^#^*p*＞0.05, ***p* < 0.01, n = 3).

**Supplementary Figure S4. NGS analysis of the DEGs affected by COL6A1 knockdown.**

Next-generation sequencing (NGS) was performed on HTR8/SVneo cells transfected with either siCOL6A1 1 or a negative control (NC) (n = 3) to examine changes in the mRNA expression profile following COL6A1 knockdown. Differentially expressed genes (DEGs) between the siCOL6A1 1 and NC groups are illustrated in a volcano plot.

**Supplementary Figure S5. Quantification of Figure 4**

1. Quantification of the protein levels of APJ and COL6A1 in Figure 4E.
2. Quantification of the protein levels of APJ and COL6A1 in Figure 4F.

(Data are mean ± SD, ***p* < 0.01, ****p* < 0.001, n = 3).

**Supplementary Figure S6. Quantification of Figure 6**

1. Quantification of protein levels of p-mTOR, p-AKT and COL6A1 in Figure 6A.
2. Quantification of protein levels of p-mTOR, p-AKT and COL6A1 in Figure 6B.

C. Quantification of protein levels of p-mTOR, p-AKT and COL6A1 in Figure 6C.

D. Quantification of Figure 6D.

E. Quantification of Figure 6E.

(Data are mean ± SD, **p* < 0.05, ***p* < 0.01, ****p* < 0.001, n = 3).

**Supplementary Figures：**

**Supplementary Figure S1**

**
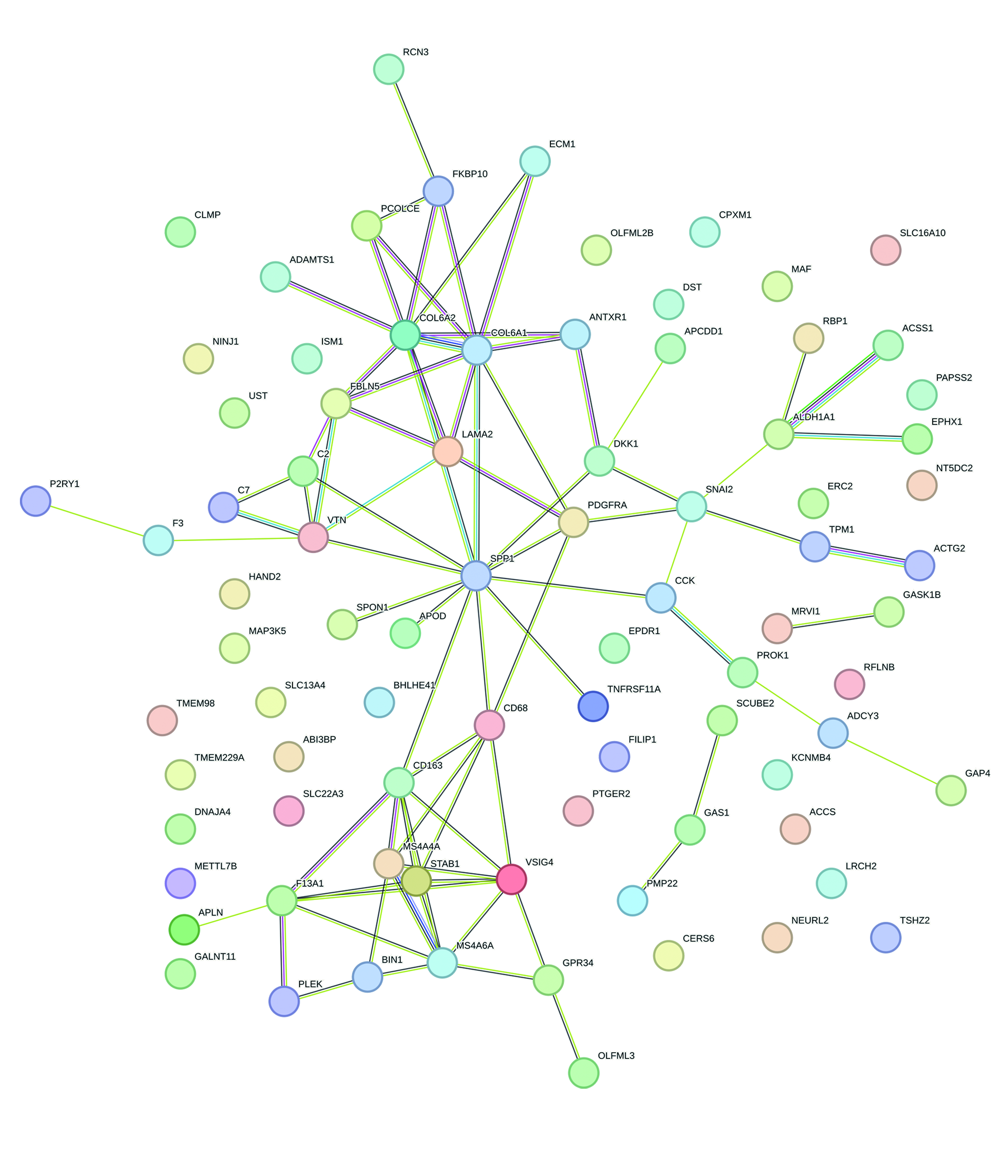
**

**Supplementary Figure S2**

**
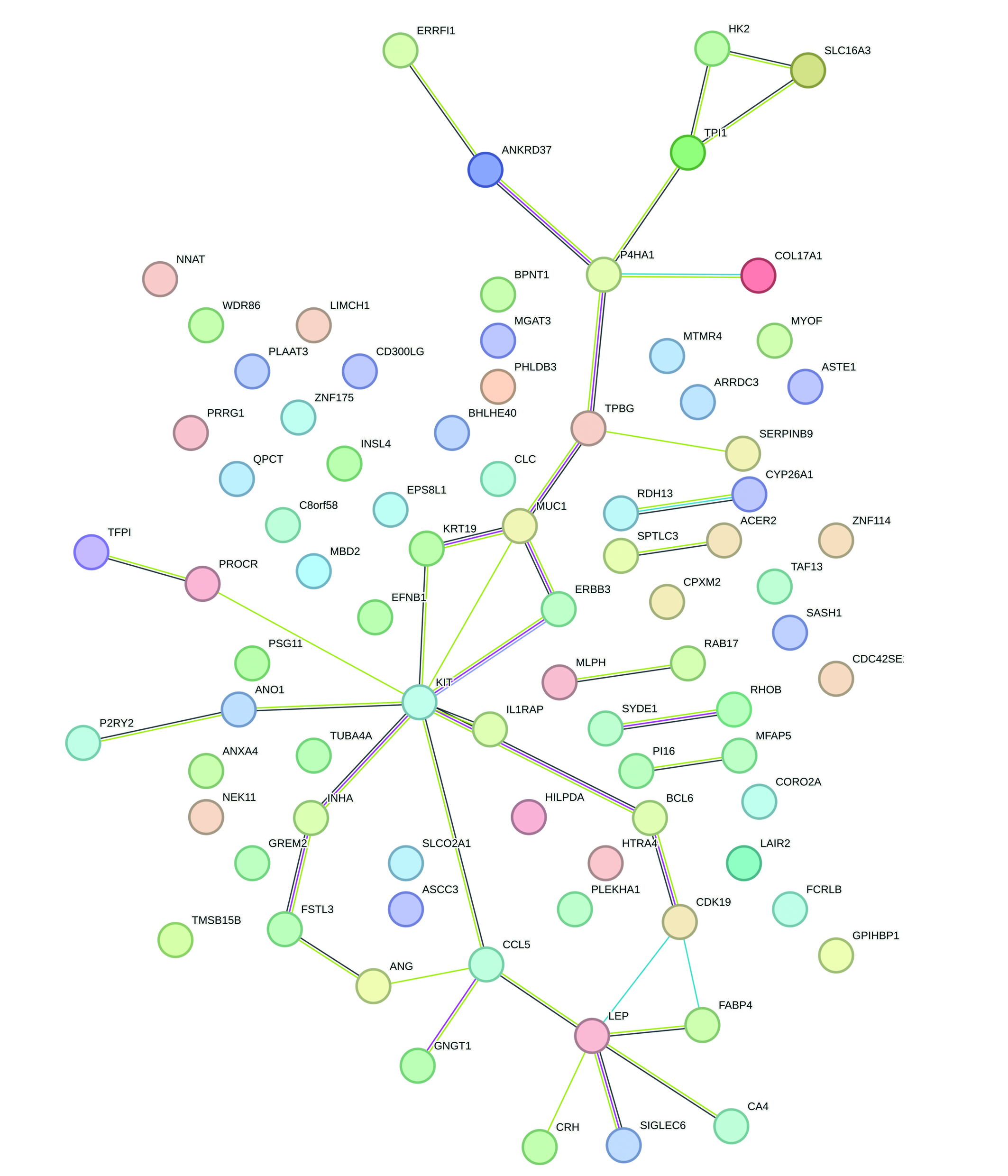
**

**Supplementary Figure S3**

**
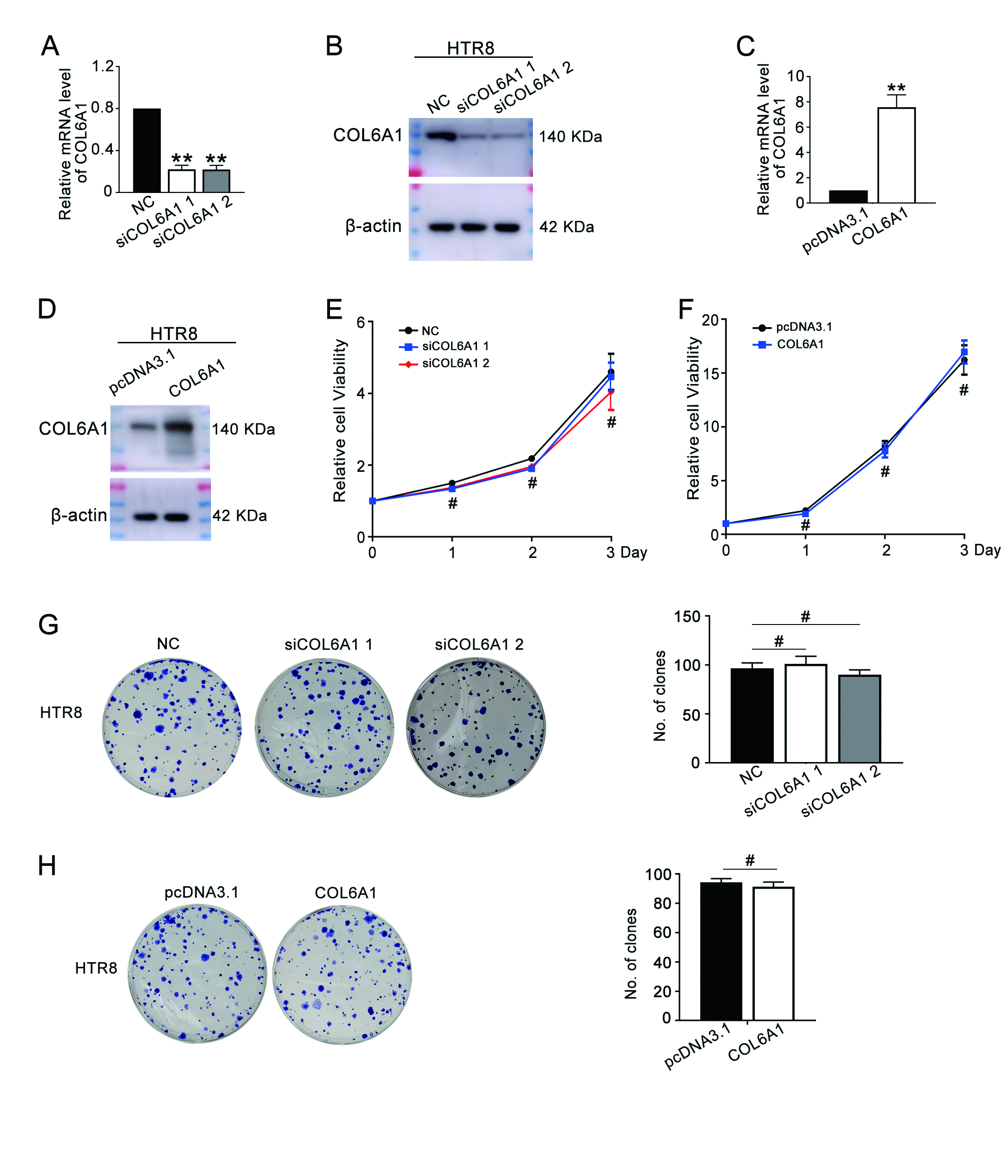
**

**Supplementary Figure S4**

**
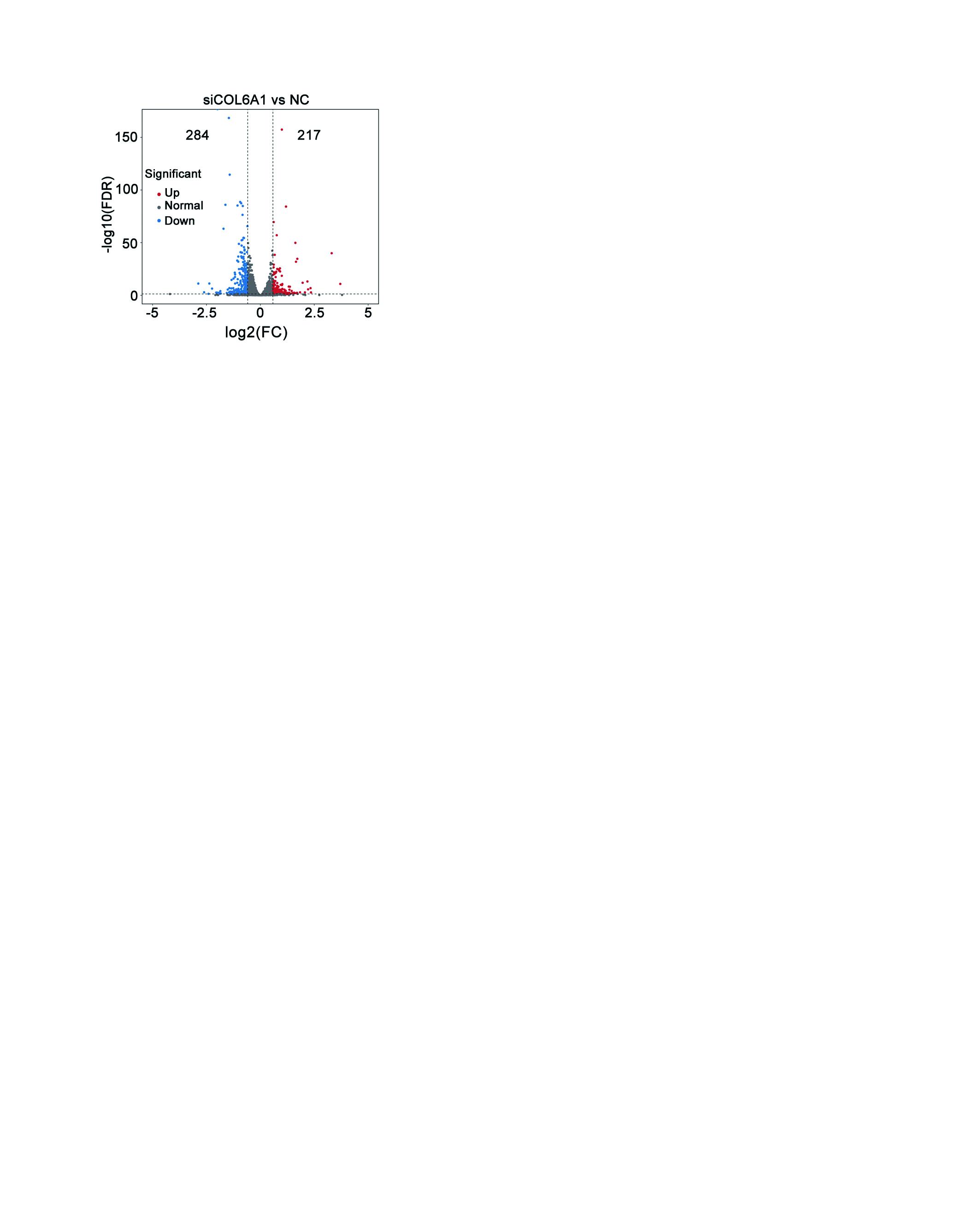
**

**Supplementary Figure S5**

**
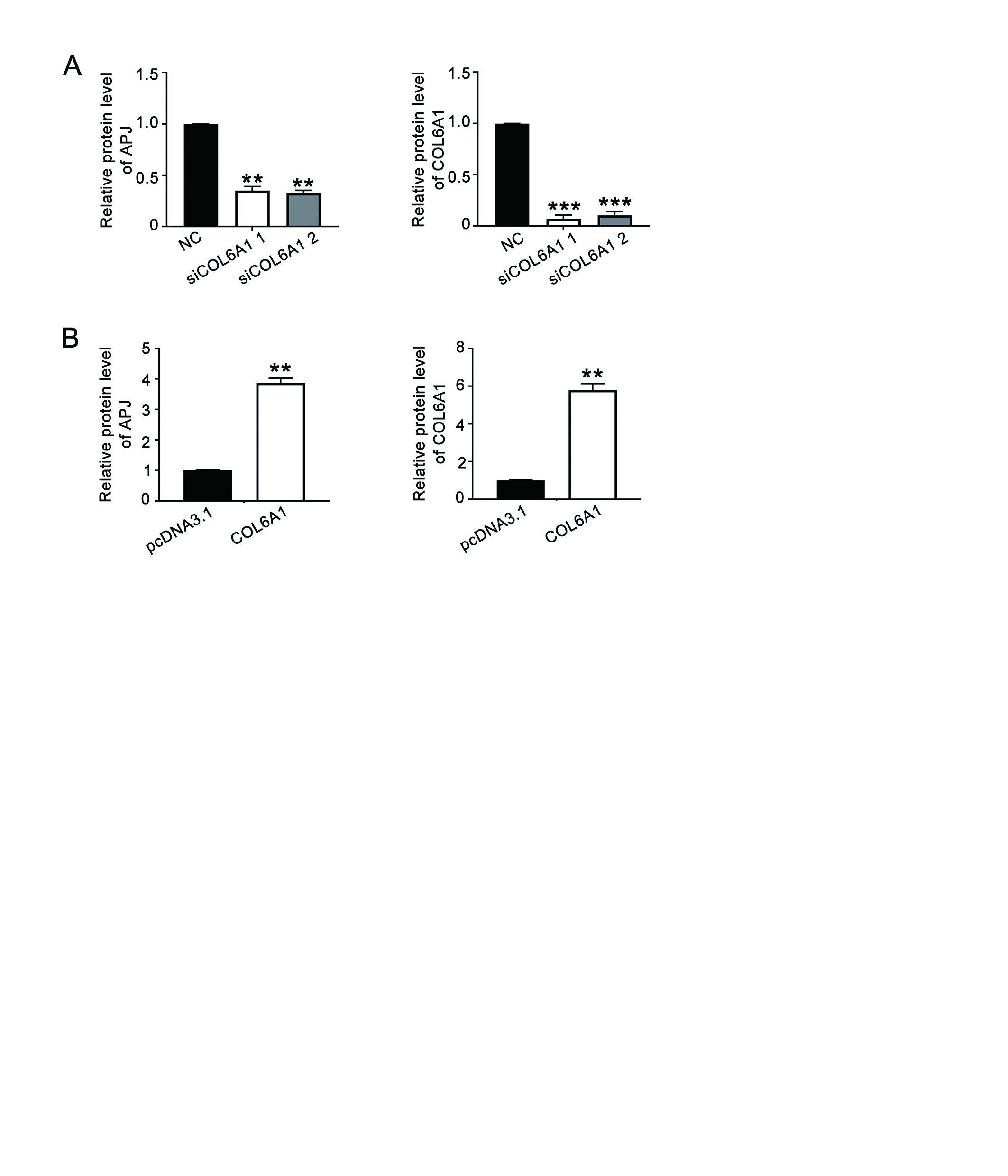
**

**Supplementary Figure S6**

**
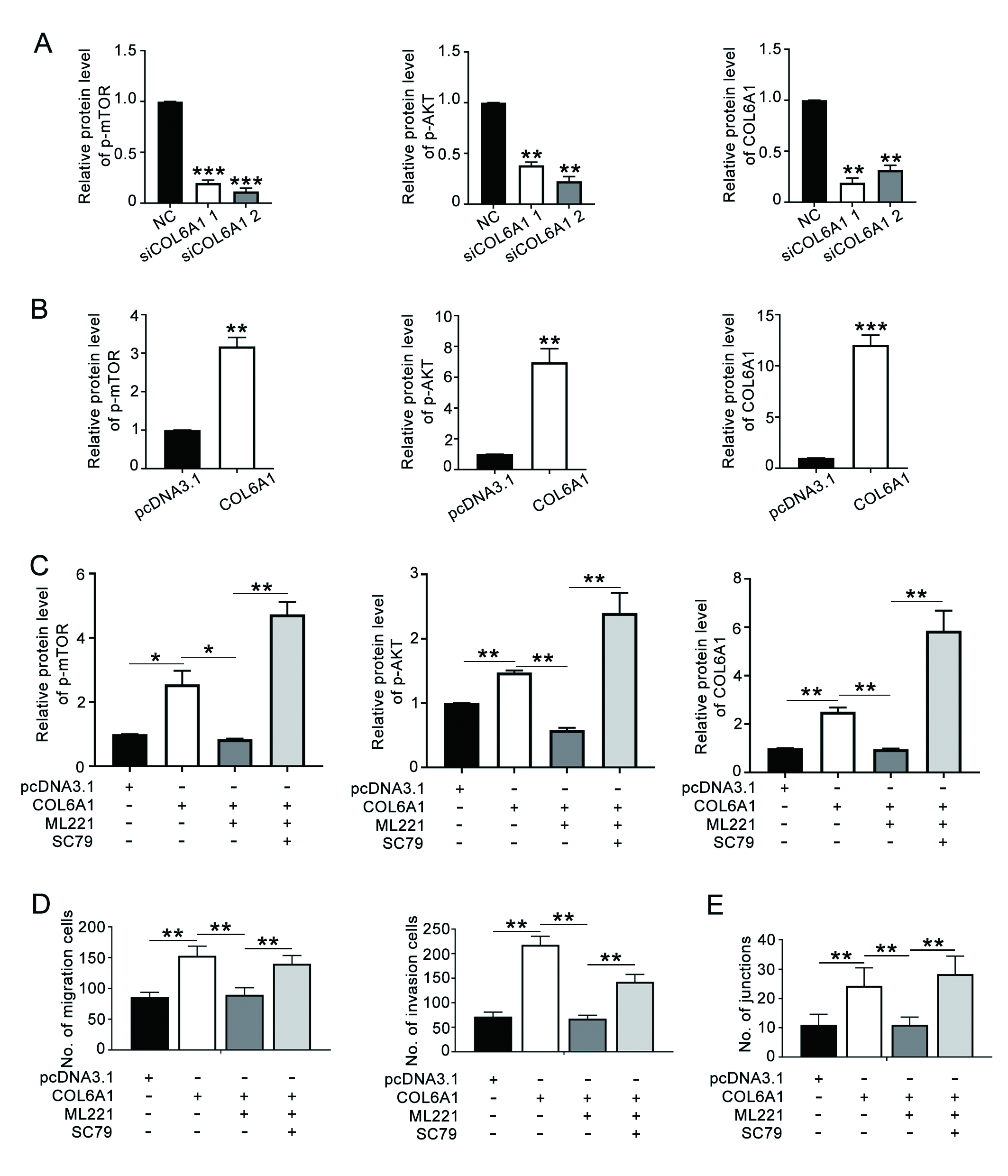
**

1. Gonghua Q, Chenyi Z, Hanlin M, Yingwei L, Jiali P, Jingying C, et al. CDCA8, targeted by MYBL2, promotes malignant progression and olaparib insensitivity in ovarian cancer. 2021;11(2).
